# Functional Deficits in nNOSμ-Deficient Skeletal Muscle: Myopathy in nNOS Knockout Mice

**DOI:** 10.1371/journal.pone.0003387

**Published:** 2008-10-13

**Authors:** Justin M. Percival, Kendra N. E. Anderson, Paul Gregorevic, Jeffrey S. Chamberlain, Stanley C. Froehner

**Affiliations:** 1 Department of Physiology and Biophysics, University of Washington, Seattle, Washington, United States of America; 2 Department of Neurology, University of Washington, Seattle, Washington, United States of America; 3 Department of Biochemistry, University of Washington, Seattle, Washington, United States of America; 4 Senator Paul D. Wellstone Muscular Dystrophy Cooperative Research Center, University of Washington, Seattle, Washington, United States of America; Hospital Vall d'Hebron, Spain

## Abstract

Skeletal muscle nNOSμ (neuronal nitric oxide synthase mu) localizes to the sarcolemma through interaction with the dystrophin-associated glycoprotein (DAG) complex, where it synthesizes nitric oxide (NO). Disruption of the DAG complex occurs in dystrophinopathies and sarcoglycanopathies, two genetically distinct classes of muscular dystrophy characterized by progressive loss of muscle mass, muscle weakness and increased fatigability. DAG complex instability leads to mislocalization and downregulation of nNOSμ; but this is thought to play a minor role in disease pathogenesis. This view persists without knowledge of the role of nNOS in skeletal muscle contractile function *in vivo* and has influenced gene therapy approaches to dystrophinopathy, the majority of which do not restore sarcolemmal nNOSμ. We address this knowledge gap by evaluating skeletal muscle function in nNOS knockout (KN1) mice using an *in situ* approach, in which the muscle is maintained in its normal physiological environment. nNOS-deficiency caused reductions in skeletal muscle bulk and maximum tetanic force production in male mice only. Furthermore, nNOS-deficient muscles from both male and female mice exhibited increased susceptibility to contraction-induced fatigue. These data suggest that aberrant nNOSμ signaling can negatively impact three important clinical features of dystrophinopathies and sarcoglycanopathies: maintenance of muscle bulk, force generation and fatigability. Our study suggests that restoration of sarcolemmal nNOSμ expression in dystrophic muscles may be more important than previously appreciated and that it should be a feature of any fully effective gene therapy-based intervention.

## Introduction

Nitric oxide (NO) is a versatile signaling molecule in skeletal muscle and is synthesized from oxygen and L-arginine by muscle-specific neuronal nitric oxide synthase mu (nNOSμ) [1], [2]. Functions of NO in muscle include: attenuation of muscle force generation and regulation of appropriate blood and oxygen delivery to active muscles during exercise [1], [3]–[7]. However *in vitro* studies of the role of nitric oxide in contractile function of excised muscles in perfusion baths have generated conflicting results. NO has been reported to increase force-generating capacity of skeletal muscle in some studies and decrease it in others [1], [3]–[5]. This has led to questioning of the physiological relevance of these *in vitro* studies [5]. These data suggest that the effects of nNOS on the force-generating capacity of muscle *in vivo* remain to be determined.

Particular interest in nNOSμ function in skeletal muscle arises from studies of human muscular dystrophies. nNOSμ is localized to the sarcolemma by interaction with the dystrophin-associated glycoprotein (DAG) complex [8], [9]. Disruption of the DAG complex results in decreased nNOSμ expression and aberrant localization. DAG complex disruption occurs in several distinct dystrophies, including Duchenne Muscular Dystrophy (DMD), Becker Muscular Dystrophy and Limb Girdle Muscular Dystrophies (LGMD) 2C, 2D and 2E [8], [10], [11]. These muscle diseases vary in severity and are characterized by progressive loss of muscle bulk, weakness and increased susceptibility to fatigue. Each disease is characterized by defects in nNOSμ expression and/or targeting. Indeed, DMD patients exhibit defective inhibition of vasoconstriction during exercise causing functional muscle ischemia that may exacerbate dystrophic muscle damage [12], [13]. These studies suggest that loss of nNOSμ may contribute to disease pathogenesis.

Although aberrant nNOSμ localization and expression is a feature of the pathology of DMD, BMD and several LGMDs, it is not known whether the loss of nNOSμ can cause contractile deficits in normal or dystrophic muscle *in vivo*. Indeed, *in vitro* studies of NO regulation of muscle contractility suggest that nNOSμ-deficiency may actually enhance the force generating capacity of skeletal muscle [3], [4]. This thinking has influenced the development of gene-therapy based therapeutic approaches to treating dystrophin-deficient muscles of DMD patients. Viral-mediated delivery of micro- or mini-dystrophin constructs substantially improves dystrophic pathology without restoring nNOSμ expression at the sarcolemma in the *mdx* mouse model of DMD [30], [31]. Whether this is a significant limitation of the gene-therapy-based approach remains to be established. It could be a significant limitation if nNOSμ-deficient skeletal muscles exhibit functional deficits *in vivo*.

In order to determine if the absence of nNOSμ negatively impacts skeletal muscle function, we evaluated the force-generating capacity *in situ* of tibialis anterior (TA) muscles from nNOS knockout (KN1) mice. Given the reported effects of nNOSμ on blood supply during exercise, it was important to use an *in situ* approach where the TA muscle was maintained in the most physiologically relevant state with normal vascularization. Unexpectedly, nNOSμ-deficient muscles from male mice were smaller in mass and generated significantly lower maximum isometric force compared with littermate controls. Moreover, muscles from both male and female mice lacking nNOSμ show increased susceptibility to fatigue compared with controls. In contrast to previous *in vitro* studies, our data suggest that nNOSμ-deficiency results in reduced force-generating capacity and that NO is necessary for sustained muscle contractility. These data also suggest the possibility that mini- and micro-dystrophins capable of restoring sarcolemmal nNOSμ expression may be more effective at reversing the functional deficits of dystrophic skeletal muscle. The combination of reduced bulk and impaired contractile function lead us to conclude that nNOSμ-deficient muscles are myopathic and that aberrant nNOSμ expression could contribute to functional deficits, especially increased susceptibility to fatigue, in DMD, BMD and LGMD 2C, 2D and 2E. We propose that the *NOS1* gene be considered a novel candidate for skeletal muscle myopathies.

## Results

### Sex-Specific Decrease in Skeletal Muscle Bulk of nNOS-Deficient Mice

**Figure 1:**
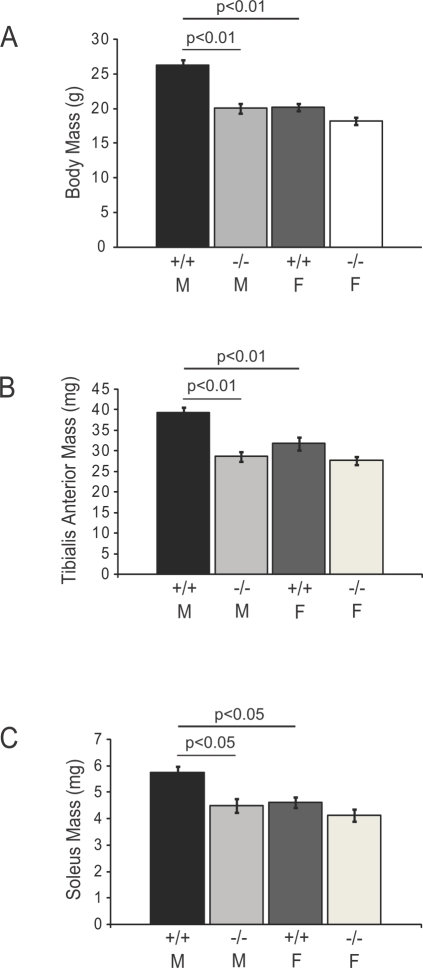
Sex-specific reductions in body and skeletal muscle mass in nNOS-deficient mice. The body masses of 8 week old adult male (M) and female (F) mice homozygous for the wild type *NOS1* allele (+/+) or *NOS1* null allele (−/−) are shown in A. The body masses of wild type males are significantly larger (p<0.01) than KN1 littermates and wild type females (p<0.01) (A). The masses of tibialis anterior (TA) muscles from 8 week old adult wild type males are significantly larger (p<0.01) than KN1 male littermates and wild type females (B). The masses of soleus muscles are similarly affected in nNOS mutant mice where KN1 male solei are significantly smaller (p<0.05) than male littermate controls (C). The soleus muscle of wild type males is larger than that of wild type females (p<0.05). Numbers of animals analyzed: 10–11 wild type males, 11 KN1 males, 10–11 wild type females and 12–13 KN1 females. Values shown are mean±standard error of the mean (S.E.M).

*Prima facie*, the most striking phenotypic characteristic of the nNOS mutant KN1 mice was the reduced body mass of males. Wild type males were on average 6 g heavier than their sex- and age-matched KN1 littermates (Figure 1A). Heterozygous mice appeared similar to wild type mice (data not shown). The well-established sexual dimorphism in body weight was evident from the significantly larger body masses of wild type males relative to wild type females. In contrast, the absence of nNOS did not impact the body mass of KN1 females (Figure 1A). Male mice are unable to achieve normal size in the absence of nNOS.

Since, skeletal muscle mass accounts for approximately 40–50% of the total body mass of a normal male 8 week old mouse, reductions in skeletal muscle weight could account for the decreased size of the KN1 males. In order to determine the effect of nNOS-deficiency on muscle mass, the wet weights of two hindlimb muscles, the tibialis anterior (a predominantly fast twitch muscle) and soleus muscles (a predominantly slow twitch muscle), were measured. The masses of both the TA (Figure 1B) and soleus muscles (Figure 1C) were significantly decreased in male nNOS mutant mice only, compared with wild type male littermates. Mass reduction occurs in skeletal muscles with different fiber compositions, and therefore could reasonably account for the reduced body mass. In contrast, in female mice, the masses of both TA and soleus muscle were not significantly affected by the absence of nNOS (Figure 1B and 1C, respectively). Together, these data suggest the possibility that nNOS can act as a sex-specific regulator of skeletal muscle mass in mice.

### Decreased Maximum Tetanic Force-Generating Capacity of nNOSμ-Deficient Skeletal Muscle

**Figure 2:**
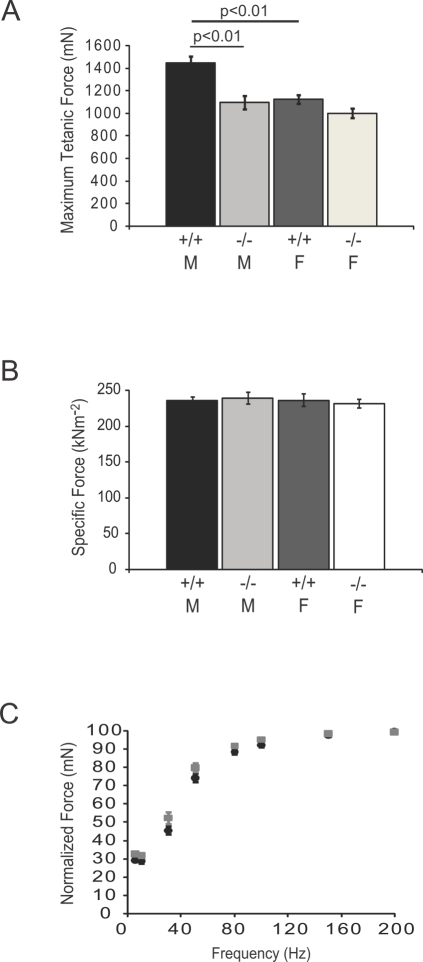
Sex-specific decrease in maximum isometric force-generating capacity in nNOSμ-deficient skeletal muscle. Maximum isometric force output (P_o_) from TA muscles of 8 week old adult male (M) and female (F) mice homozygous for the wild type *NOS1* allele (+/+) or or *NOS1* null allele (−/−) is shown in A. P_o_ was significantly decreased (p<0.01) in male KN1 TA muscle compared with sex-matched littermate controls (A). Wild type male mice generated greater total force than wild type female mice (p<0.01). Maximum tetanic force output from female TA skeletal muscle was unaffected by the absence of nNOSμ (A). Specific force (sP_o_), maximal tetanic force output normalized for the length and mass of the TA muscle, was not significantly affected by the absence of muscle nNOSμ in both males and females (B). Force-frequency profiles for wild type (black circles) and KN1 mice (grey squares) are shown in C. Males and females are pooled into wild type and KN1 groups. There was no difference in the force-frequency curves between KN1 males and their littermate controls suggesting no gross abnormalities in neuromuscular transmission. Numbers of animals analyzed: 15 wild type (8 males and 7 females) and 15 KN1 (7 males and 8 females). Values shown are mean±standard error of the mean (S.E.M).

The amount of contractile force or tension a given skeletal muscle generates is generally proportional to the mass and length of the muscle itself. Given the reduced TA muscle bulk in male KN1 mice, we tested whether the maximum tetanic force-generating capacity of nNOSμ-deficient TA muscles was also decreased (Figure 2). Maximum tetanic force was significantly decreased in KN1 male TA muscles only (Figure 2A), paralleling the decreases in muscle mass described above. The maximum force-generating capacities of KN1 males, wild type females and KN1 females were not significantly different (Figure 2A). Wild type male TA muscles generated larger tetanic force than those from wild type females. In summary, male nNOSμ-deficient TA muscles are significantly weaker than littermate controls.

To investigate whether deficits in force production in male KN1 mice could be accounted for by decreased TA mass, we calculated specific force (sP_o_), the maximal tetanic force output normalized for the cross sectional area of the TA muscle. sP_o_ was not significantly affected by the absence of muscle nNOSμ in either sex (Figure 2B). These data support the argument that decreased force generation in male KN1 TA muscles is most likely due to decreased muscle mass and not to defects in the contractile apparatus.

nNOS has been reported to regulate the structure and function of the neuromuscular synapse [15]. In order to evaluate the possibility that abnormalities in neuromuscular transmission were contributing to the maximal tetanic force deficit, we evaluated force-frequency curves from wild type and KN1 control males. No significant difference between control and wild type males was found (Figure 2C), suggesting no gross abnormalities in neuromuscular transmission. This is consistent with previously published results where the absence of nNOSμ from postsynaptic folds had only a minor effect on neuromuscular junction transmission [15]. Taken together, the decreased maximum force and unchanged specific force output suggest that the sex-specific reduction in maximal tetanic force in nNOSμ-deficient muscle is most likely due to reduced muscle mass.

### nNOSμ-Deficient Skeletal Muscle Exhibit Increased Susceptibility to Contraction-Induced Fatigue

**Figure 3:**
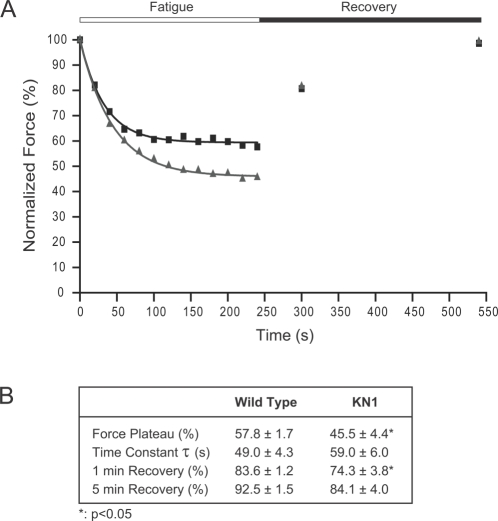
nNOSμ-deficient skeletal muscle exhibits decreased resistance to exercise-induced fatigue. To simulate exercise, TA muscles were maximally stimulated every 2 s for 4 minutes (fatigue period). Force recovery was measured after 1 minute and 5 minutes of rest following the conclusion of the fatigue period. Representative data are shown for wild type (black boxes) and KN1 (grey triangles) and are fitted with exponential decay curves (black for wild type and grey for KN1) (A). Both male and female KN1 mice showed similar patterns of fatigue. During 4 minutes of repeated contraction, nNOSμ-deficient skeletal muscles did not sustain control levels of force (A). Force generation capacity of KN1 mice declined to a significantly lower force plateau (p<0.05) than wild type controls (B). The time taken to fatigue represented by the time constant τ, was not significantly different between wild type and KN1 mice (B). After 1 minute of rest, nNOS-deficient TA muscles did not recover to the same extent as wild type TA muscle (p<0.05). However, after 5 minutes of rest, the force-generating capacity of nNOS-deficient muscles was fully recovered (B). Numbers of animals analyzed: 13 wild type (7 males and 6 females) and 13 KN1 (5 males and 8 females) mice. Values shown in B are mean±standard error of the mean (S.E.M).

We then tested the hyopthesis that nNOS deficiency impacts sustained force generation during muscle activity, i.e., the absence of nNOS may decrease resistance to exercise-induced fatigue. In order to test this possibility, TA muscles from KN1 mice were subjected to a simulated exercise protocol in *situ* (see Methods). Representative data from wild type and KN1 TA muscles are shown in Figure 3A and the averages are presented in 3B. After 40 s of simulated exercise, nNOSμ-deficient muscles begin to show deficits in force production (Figure 3A). By the end of the exercise protocol, the force-generating capacity of KN1 muscle had declined to a significantly lower force plateau (averaging 45.5±4.4%) compared with littermate controls (57.8±1.7%), (Figure 3B). Note that the trace shown exhibited near normal recovery. These data highlight a marked increase in fatigability of nNOSμ-deficient skeletal muscle. The time taken to fatigue, as represented by the time constant τ, is not significantly different between nNOSμ-deficient TA muscles and controls (Figure 3B). After a 1 minute recovery period, KN1 muscles remain unable to generate normal levels of force compared with controls (Figure 3B). At 5 minutes post-exercise, nNOS-deficient muscles exhibit a full recovery (Figure 3B). Both male and female KN1 TA muscles showed similar enhanced susceptibility to exercise-induced fatigue (data not shown). It is important to note that susceptibility to fatigue was not simply a consequence of reduced muscle mass. TA masses from wild type female mice, male KN1 and female KN1 mice did not differ significantly (Figure 1B), but only nNOS-deficient TAs were more fatigable. These data highlight a novel role for nNOS in regulating sustained force generation during muscle activity.

Next, we addressed potential mechanism(s) of decreased fatigue resistance in nNOSμ-deficient skeletal muscle. We hypothesized that susceptibility to fatigue could be due to increased numbers of fast twitch fibers and fewer slow twitch fibers. Fast twitch fibers have a more glycolytic metabolism and thus fatigue more easily than slow twitch myofibers. A greater proportion of fast twitch fibers would decrease the time taken for the muscle to reach peak twitch tension (P_t_) and shorten the relaxation phase. The twitch responses of nNOS-deficient muscles to a single threshold stimulus are shown in Table 1. Peak twitch force (P_t_) was significantly reduced in KN1 males compared with controls. Despite this decrease, nNOSμ-deficient muscles did not exhibit any intrinsic weakness, because specific twitch force (sP_t_, [P_t_ normalized for cross sectional area]) did not differ from littermate controls (Table 1). However, in contrast to male KN1 muscle, P_t_ was unaffected by the absence of nNOSμ in female mice (Table 1). KN1 male mice exhibit a sex-specific decrease in Pt, paralleling the decreased TA muscle mass and maximum isometric force deficit. The loss of nNOSμ from skeletal muscle had no significant impact on the time to reach peak tension and the time between maximum and half-maximum force production during the relaxation phase of the twitch (Table 1). The absence of significant difference in the time of the contraction and relaxation phase of the twitch suggest that there is no gross change in the fiber composition of the TA muscle and argue against a fiber type shift as a mechanism for the increased fatigability of nNOSμ-deficient muscle.

**Table 1:** Twitch Properties of nNOSμ-Deficient Skeletal Muscle Fibers.

	*Male*		*Female*	
	*Wild Type*	*KN1*	*Wild Type*	*KN1*
P_t_ (mN)	486±22	376±17‡	373±19#	350±13
TPT (ms)	17.8±1.5	20.2±1	15.7±0.6	19.2±0.9
HRT (ms)	19.5±1.4	22.6±1.1	19.1±1.7	22.7±1.6
sP_t_ (kNm^−2^)	79.4±3.2	80.7±3	77.1±3.6	78.4±2.9

P_t_ is twitch force. TPT is the time taken to reach peak tension during the contraction phase of the twitch. HRT is the time between maximum and half-maximum force generation during the relaxation phase of the twitch. sP_t_ is specific twitch force. Mean values±SEM are shown. ‡ p<0.01, for comparison between wild type and KN1 males. # p<0.01, for comparison between wild type males and females.

**Figure 4:**
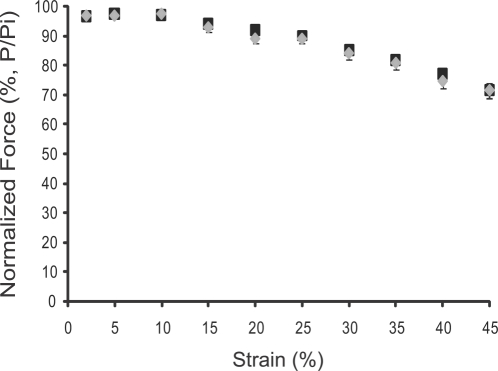
nNOSμ-deficiency does not affect the susceptibility of the TA to contraction-induced injury. Contraction-induced injury was produced by subjecting TA muscles to a series of consecutive lengthening contractions of progressively increasing strain. Strain is the percentage increase in length beyond optimal muscle length Lo. Contractile function is expressed as normalized force (force/initial force or P/Pi). The susceptibility to contraction-induced injury for wild type (black squares) and nNOS-deficient (grey diamonds) TA muscles are shown. There was no significant difference between controls and KN1 littermates. Numbers of animals analyzed: 14 wild type (8 males and 6 females) and 11 KN1 (5 males and 6 females) mice. Values shown are mean±standard error of the mean (S.E.M).

An alternative possibility was that the decreased resistance to fatigue in KN1 skeletal muscle was caused by an increase in the TA muscle's susceptibility to contraction-induced injury. Therefore we tested whether nNOSμ-deficiency impacts resistance to contraction-induced injury *in situ* by subjecting TA muscles to a series of consecutive lengthening contractions of progressively increasing strain (Figure 4). We observed no significant difference in resistance to contraction-induced injury between wild type and nNOSμ-deficient TA muscles over a wide range of strains (Figure 4). Thus, the absence of nNOSμ does not affect the susceptibility of the TA to contraction-induced injury. The impaired performance of nNOSμ-deficient skeletal muscle during exercise is unlikely to be due to an increased predisposition to contraction-induced injury.

## Discussion

This study provides surprising new insights into nNOSμ function in skeletal muscle *in vivo*. nNOSμ-deficiency significantly reduces skeletal muscle mass, maximum tetanic force output and fatigue resistance; therefore qualifying the skeletal muscles of KN1 mice as myopathic. Unexpectedly, myopathy is most severe in nNOS-deficient male mice, because female mice exhibit increased fatigability only. Despite the *NOS1* gene being localized to murine autosome five, and not on a sex chromosome, the impact of nNOS on skeletal muscle is mediated by unknown sex-specific mechanisms. This emphasizes the importance of considering gender when experimentally assessing nNOS function. Our data suggest that nNOSμ is an important regulator of skeletal muscle size and contractile performance.

One of the key features of nNOSμ-deficient skeletal muscle is increased susceptibility to fatigue, arguably the most significant new finding of the present study. The force generated by nNOSμ-deficient muscles during simulated exercise plateaus at a significantly lower level than controls (Figure 3B). Recovery appears to occur more gradually in KN1 mice; however there is full restoration of force-generating capacity after a five minute rest period. In order to address potential mechanisms of decreased resistance to fatigue in KN1 mice, we looked for functional evidence of increased numbers of fast twitch fibers in KN1 TA muscle (Table 1). Fast twitch fibers are more easily fatigued than slow twitch fibers. Contraction and relaxation times of single muscle twitches were unaffected by the absence of nNOSμ, arguing that increased fatigability was not due to alterations in the ratio of fast to slow twitch fibers. nNOSμ-derived NO plays an important role in maintaining normal blood supply during exercise by overriding α-adrenergic receptor-mediated vasoconstriction [6], [7]. Impaired blood and oxygen delivery to the vascular beds of active muscles can result in repeated muscle exposure to functional ischemia [12]. Therefore we speculate that decreased muscle oxygenation could account, at least in part, for the susceptibility to fatigue of nNOSμ-deficient TA muscles. Additional evidence to support this possibility comes from dog studies *in vivo* where pharmacological inhibition of nitric oxide synthesis was proposed to cause fatigue by mismatching blood supply with demand in contracting skeletal muscles [18]. Muscle fatigue is a complex physiological process that can occur by many different mechanisms, further studies are required to address the exact mechanism(s) by which nNOS-deficiency results in increased susceptibility of skeletal muscle to fatigue. At present, nNOSμ deficiency does not appear to increase fatigability by significantly altering TA muscle fiber type composition, but may do so through impaired vasoregulatory mechanisms.

NO has been previously suggested to be an inhibitor of the force-generating capacity of skeletal muscle. In *in vitro* bath studies on excised muscles, the pharmacological inhibition of NOS results in greater force output [3], [4]. Thus, nitric oxide is widely understood to act as an inhibitor or attenuator of muscle contractility. However, these data are not consistent with our findings *in vivo*. We find that the absence of nNOSμ is associated with decreased maximal force-generating capacity in males. This is most likely due to decreased muscle mass and not due to defects in the contractile apparatus function; because specific force (sP_o_) was unchanged (Figure 2B). Similarly, the mass and force-generating capacity of female TA are unaffected by the absence of nNOSμ; therefore the differences between male and female force maximal force generation are likely due to differential impact on muscle mass. Also, inconsistent with the thesis that NO attenuates muscle function, we found that force frequency curves were unaffected by the loss of nNOSμ and the maximum force-generating properties of female muscle were unaffected by the loss of nNOS. In our view, the most likely explanation of the conflicting reports of the impact of NO on contractility is provided by the different experimental approaches employed. We used an *in situ* approach where the TA muscle is kept in the most physiologically relevant environment possible, within normal physiological temperature range, with intact motor neuron innervation and vasculature [17]. This is very important for evaluating nNOSμ function in muscle due to its known vasoregulatory role. We also circumvented the limitations of pharmacological inhibition of NOS by using the KN1 mouse to specifically inactivate nNOS. Our view is supported by a recent study questioning the physiological relevance of studies designed to address the role of NO in muscle contraction that were conducted on excised muscles in physiological baths *in vitro* [5]. It was reported that regulation of muscle contractile function by nNOSμ depended dramatically on oxygen concentration [5]. At non-physiological oxygen concentrations, such as those found in excised *in vitro* muscle preparations, NO inhibited muscle contractility. Conversely, at low physiological oxygen tensions resembling those found in muscle tissue, NO increased the force-generating capacity of skeletal muscle [5]. Our finding that nNOS deficiency results in force loss *in situ* is consistent with *in vitro* studies performed under physiological oxygen conditions. Therefore, our *in situ* methods presumably maintain more physiologically relevant concentrations of oxygen in muscle tissue. In summary, these data suggest that nNOS-derived NO is necessary for maximal force-generating capacity of muscle *in vivo* and that nNOS plays an important role in the contractile performance of skeletal muscle. Furthermore, these results are consistent with a role of both oxygen and NO in coordinated regulation of the force-generating capacity of skeletal muscle.

The impact of nNOS-deficiency on muscle contractility has implications for two classes of muscle disease: metabolic myopathies and muscular dystrophies. Skeletal muscle fatigue is a common cause of weakness in human myopathies that result from congenital defects in energy metabolism [18]. These muscle diseases often elude diagnosis since symptoms occur predominantly or exclusively during exercise. Often symptoms are simply attributed to a lack of fitness [18]. Since the skeletal muscle phenotype of KN1 mice is consistent with that of human metabolic myopathies, the *NOS1* gene may be a novel candidate gene for this class of skeletal muscle disease. The deficiencies in nNOSμ-deficient skeletal muscle are also especially relevant to two types of muscular dystrophy, dystrophinopathies (DMD and BMD) and sarcoglycanopathies (LGMD 2C, LGMD 2D and LGMD 2E), two genetically distinct classes of muscular dystrophy where skeletal muscle nNOSμ expression and localization is dysregulated as a secondary consequence of the disruption of the DAG complex [8], [10], [11], [19]. nNOSμ protein expression is undetectable in cytoplasmic or plasma membrane fractions from DMD patient biopsies [19]. Aberrant nNOSμ activity may contribute to the pathogenesis in DMD and LGMD by impairing blood flow in active muscles resulting in ischemia [6], [7]. Indeed, increased NO bioavailability in mouse models of DMD (*mdx* mice) and LGMD2D (α-sarcoglycan null mice) substantially improves the pathology of dystrophic skeletal muscle tissue [20]–[22]. Our data suggest that specific characteristics of dystrophic pathology, including the inability to maintain muscle bulk, inherent muscle weakness and susceptibility to fatigue may be attributable, at least in part, to defects in nNOS signaling. Our data also raise the intriguing possibility that loss of nNOSμ is more detrimental to muscle function in male patients than in female patients.

The pervasive inherent weakness of dystrophin-deficient muscle is due to both increased susceptibility to injury by lengthening contraction and fatigue [23]–[26]. The impaired performance of α- and γ-sarcoglycan-deficient skeletal muscles is also due, at least in part, to increased susceptibility to fatigue, but not contraction-induced injury [26]–[28]. Our data argue against a role for aberrant nNOSμ expression contributing to increased susceptibility to contraction-induced injury, but do suggest the possibility that dysregulation of nNOS signaling provides a mechanism for muscle activity-induced fatigue, a contributing factor to the impaired contractile performance of human and murine dystrophic muscle [23]–[28]. These findings make nNOS signaling pathways relevant to therapeutic approaches to muscular dystrophy.

Therapeutic approaches to treating muscular dystrophies must address the progressive loss of muscle mass and extreme muscle weakness. The ideal method to do this is to replace the defective gene with a wild type copy using gene or cell therapy based approaches [14]. However, in the case of gene therapy of DMD, this is not currently possible because dystrophin is too large to be packaged into adeno-associated viral vectors. This has led to replacement strategies that involve viral vector-mediated delivery of truncated “designer dystrophin” constructs in DMD [14]. These micro- and mini-dystrophins cause substantial improvements, but do not fully restore the dystrophic pathology and muscle function in *mdx* mice. Importantly, these mutant dystrophins do not restore nNOSμ [14]. Although the substantial improvement of dystrophic muscle function by mini- and micro-dystrophin suggests a minor role for nNOS in *mdx* pathology; the evaluation of fatigue is rarely, if ever, assayed in treated muscles despite reports of fatigue in both mice and patients [23]–[25]. Furthermore, *mdx* mice exhibit increased muscle mass, in contrast to Duchenne patients who suffer progressive loss of muscle mass. The present study suggests that the ability to restore nNOSμ would likely confer additional important functionality to the designer dystrophins. These data strongly argue that the restoration of sarcolemmal nNOSμ in dystrophic muscle may be more important than previously appreciated.

## Materials and Methods

### nNOS-Deficient Mice

B6.129S4-*Nos1* ^tmplh^/J mice purchased from The Jackson Laboratory, ME, USA were used to establish a colony. These mice are commonly known as nNOS knockout or KN1 mice and were generated by targeted disruption of exon 2 of the *NOS1* gene [29]. Exon 2 encodes the PDZ domain of nNOSα and muscle–specific nNOSμ; therefore, the skeletal muscles of KN1 mice lack nNOSμ. All comparisons are made between sex- and age-matched littermates.

### *In Situ* Contractile Function Analyses

All experimental procedures performed on mice were approved by the Institutional Animal Care and Use Committee of the University of Washington. We performed *in situ* analysis of TA muscle function with modifications as described previously [17]. This approach allows for measurement of contractile properties without removing the muscle from its natural environment, thereby maintaining normal vasoregulation and innervation. Eight week old mice were anesthetized with intraperitoneal injections of 2,2,2, tribromoethanol (Sigma, St Louis, MO). Mouse hindlimbs were shaved and the distal TA tendon of the tibialis anterior (TA) muscle was surgically isolated via a skin incision on the anterior surface of the lower hindlimb. The mouse was positioned on a 37°C heated platform in order to restrain the knee joint and the distal tendon was attached to the lever arm of a servomotor (Model 305B-LR, Aurora Scientific, ON, Canada). The exposed surface of the muscle was kept moist by frequent application of prewarmed isotonic saline. The TA muscle was stimulated by electrical trigger of the peroneal nerve using two needle electrodes. The muscle was adjusted to an optimum length (L_o_) that produced the maximum twitch force (*P*_t_). Then, the time to reach peak tension (TPT) during the contraction phase of the twitch, and the half-relaxation time (HRT), the time between maximum and half-maximum force during the relaxation phase of the twitch were recorded. While held at L_o_ the TA was stimulated every two minutes at increasing frequencies (5 to 200 Hz) to generate force-frequency curves. Maximal tetanic force (P_o_) generation was typically achieved at 200 Hz. After the completion of testing, both L_o_ and TA mass were recorded and used to normalize P_t_ and P_o_ for TA muscle size and calculate specific twitch (sP_t_) or specific tetanic (sP_o_) force. The first hindlimb was used for testing resistance to exercise-induced fatigue, while the second was used to test susceptibility to contraction-induced injury.

### Resistance to Exercise-Induced Fatigue

To test the capacity of muscle to sustain force output, TA muscles were subjected to a series of repeated contractions to simulate exercise and cause fatigue. Muscles were subject to maximal stimulation (40 V, 200 Hz) at 2 s intervals for 4 minutes. Maximum isometric force production was recorded every 2 s. Recovery from fatigue was assessed by recording P_o_ at 1 minute and 5 minutes after the completion of the fatigue period. Exponential curves (y = Ae^−x/τ^ where A is the initial force (mN), x is time (s) and τ is the time constant) were fitted to the 4 minute fatigue period with Igor Pro 5 software (Wavemetrics, OR). The time constant τ (reflecting the timecourse of fatigue in seconds where a larger τ value represents a slower rate of fatigue) and the force plateau (the asymptote of the exponential curve) were calculated from these curves.

### Contraction-Induced Injury

The resistance of muscles to contraction-induced injury was assessed by subjecting TA muscles to a series of consecutive lengthening contractions of progressively increased strain. Strain is the percentage increase in length beyond the optimal muscle length L_o_. Muscles were maximally stimulated (4 V, 200 Hz) for 150 ms at fixed length to achieve maximal isometric tension, immediately followed by 200 ms of stimulation during the application of a length change ranging from 0 to 45% beyond Lo. Strain was applied at the rate of 2 fiber lengths/s. Lengthening contractions were performed at 30 s intervals to minimize the impact of fatigue on force-generating capacity. The maximum tetanic force generated immediately prior to the initiation of the subsequent lengthening contraction was recorded and normalized. At the conclusion of contractile function analysis, animals were sacrificed and the tibialis anterior and soleus muscles were rapidly excised and weighed.

### Statistical Analyses

The number of animals of each sex and genotype analyzed for each experimental condition are given in the figure legends. All values are reported as mean±SEM. Two way univariate analysis of variance (ANOVA) was used to determine the statistical significance of the effects of gender and genotype on experimental measures using SPSS software (SPSS Inc.). For all other comparisons, values were compared using unpaired Student's *t*-tests using Microsoft® Excel 2007 software. *p* values less than 0.05 were considered significant.
